# Mediation Analysis of the Relationship Between Health Literacy and the French General Population’s Opinions on Hepatitis B Vaccination: Representative Cross-Sectional Survey of the SLAVACO Project

**DOI:** 10.2196/82496

**Published:** 2026-02-13

**Authors:** Bakary Cissé, Sylvie Boyer, Jeremy K Ward, Julien Mancini

**Affiliations:** 1 Aix Marseille Univ, INSERM, IRD, ISSPAM, SESSTIM, Sciences Economiques & Sociales de la Santé & Traitement de l’Information Médicale Marseille France; 2 CERMES3 (INSERM, CNRS, EHESS, Université de Paris) Paris France; 3 APHM, Hop Ste Marguerite, BioSTIC, Biostatistique et Technologies de l’Information et de la Communication Marseille France

**Keywords:** health literacy, hepatitis B vaccination, vaccine hesitancy, trust, SLAVACO survey, France

## Abstract

**Background:**

In France, reluctance toward hepatitis B vaccination remains high, despite the availability of a safe and effective vaccine to prevent this infection. To boost vaccination coverage, it is therefore essential to identify the factors that are likely to encourage a more favorable opinion of this vaccine. Health literacy (HL) is one such factor. It refers to the individual ability to access, understand, critically appraise, and apply health information to make informed decisions about health issues for oneself and for others.

**Objective:**

This study explored the mechanisms through which HL might affect opinions about hepatitis B vaccination, both directly and indirectly, by relevant factors, including opinions about vaccination in general, trust in government health agencies, and trust in medical doctors.

**Methods:**

The analysis used data from the SLAVACO-Wave 3 (Suivi Longitudinal des Attitudes à l’Égard d’un Vaccin Contre la COVID-19) survey, conducted in December 2021 among a representative sample of French adults (N=1932). Favorable and unfavorable opinions of hepatitis B vaccination were measured using a 5-point Likert scale, while HL was assessed using the HLS_19_-Q12 questionnaire (12-item general health literacy questionnaire used in Health Literacy Survey 2019-2021). A structural equation model examined the relationship between HL and hepatitis B vaccination opinions, taking into account the potential mediating role of trust in the health care system (ie, government health agencies and medical doctors).

**Results:**

Findings showed that individuals with a favorable opinion of hepatitis B vaccination (1437/1932, 74.4%) had a higher HL level than those with a negative or neutral opinion (62.6 vs 57.0, *P*<.001). The association between HL and hepatitis B vaccination opinions was fully mediated by trust in the health care system. The indirect effect of HL was estimated at 0.068 (95% CI 0.042-0.093), accounting for 52.4% (0.068/0.1297) of the total effect. This effect was particularly pronounced in people over 50 years (0.084, 95% CI 0.042-0.126, accounting for 0.084/0.1306, 64.3% of the total effect). Goodness-of-fit indicators were satisfactory.

**Conclusions:**

Enhancing HL might positively influence hepatitis B vaccination opinions and uptake through greater trust in the health care system. From a public health perspective, strategies should go beyond providing clear information and access to vaccines and actively work to strengthen trust in health care institutions and professionals. National campaigns correcting misconceptions about hepatitis B vaccination could be complemented by targeted interventions for groups most likely to hold negative opinions. Repeating this survey in the post–COVID-19 context could also reveal different trends, given evolving public perceptions of vaccines and health authorities.

## Introduction

Hepatitis B is a communicable infectious disease associated with an increased risk of cirrhosis and liver cancer [1]. In 2022, the World Health Organization (WHO) estimated that 254 million people were living with chronic hepatitis B infection and that approximately 1.1 million died as a result of the disease, mainly from cirrhosis or hepatocellular carcinoma [2]. Although safe and effective vaccines have been developed, 1.5 million new hepatitis B virus (HBV) infections occur annually worldwide [3,4]. The WHO aims to reduce the incidence of chronic hepatitis infection by 90% and the annual number of deaths due to chronic hepatitis by 65% before 2030 [5]. To achieve these targets, the organization’s guidelines [6] include systematic treatment for people with chronic HBV infection, the prevention of perinatal and infant infection through vaccination, and catch-up vaccinations for both high-risk populations (eg, injection drug users, men who have sex with men, and sex workers) and health care professionals. As of 2023, hepatitis B vaccination for infants was mandatory in 190 countries, and global coverage was estimated at 83% [7].

Hepatitis B is slightly endemic in France; in 2016, the estimated prevalence in the general population in metropolitan France was 0.3% [8]. Although this prevalence may seem low in the European context—it is higher than in Germany (0.2%) but lower than in Southern and Eastern European countries, where it varies between 0.5% and 1.5% [9]—hepatitis B remains a major public health problem due to its chronic progression and severe complications. Moreover, the burden is marked by unequal distribution, with significant socioeconomic disparities disproportionately affecting the most vulnerable populations [10]. Its impact is also reflected in mortality; from 2005 to 2020, a total of 2133 deaths occurred in patients hospitalized for related complications (cirrhosis or hepatocellular carcinoma) in metropolitan France, representing a lethality rate of 6% [11]. Beyond morbidity and mortality, hepatitis B also generates substantial direct and indirect health care costs, particularly in the stages of cirrhosis or hepatocellular carcinoma [12]. To combat this infection, starting in the late 1980s, the French National Authority for Health recommended vaccination for individuals at high risk of exposure and for all newborns. Moreover, a catch-up program for children and adolescents younger than 15 years was implemented in 1995 [13]. In the context of the WHO’s goal to eliminate viral hepatitis by 2030, primary vaccination became mandatory for all infants born on or after January 1, 2018 [14]. The French primary childhood immunization schedule recommends 3 doses: the first at 2 months of age, the second at 4 months, and a booster at 11 months [15]. Full immunization is considered complete once the 3-dose series has been administered. As the majority of HBV infections in France in adults occur through sexual transmission [16], vaccination is strongly encouraged for persons older than 18 years of age. The immunization schedule consists of 3 doses, administered at 0, 1, and 6 months [15]. According to the 2021 LaboHEP survey, which was conducted among all public and private bioanalytical testing laboratories to estimate hepatitis screening activity, the rate of positive HBV diagnosis in metropolitan France was 54/100,000, representing a 10% increase compared to 2016 [17]. In addition, although hepatitis B vaccination coverage among infants has gradually increased over time, reaching over 90% for children aged 21 months since 2018 [18], it was below 50% among adolescents and adults in 2015 [19,20]; this reflects a clear generational gap in hepatitis B immunization.

Health Barometer surveys conducted between 2010 and 2023 show that in terms of vaccine hesitancy rankings in metropolitan France, the hepatitis B vaccine placed third after COVID-19 and influenza vaccines [21]. Despite this, no recent study has investigated the determinants of hesitancy over this vaccine. In the present analysis, we formulate the hypothesis that health literacy (HL), defined by Sørensen et al [22] as a concept “linked to literacy and that entails people’s knowledge, motivation and competences to access, understand, appraise, and apply health information to make judgments and take decisions in everyday life concerning healthcare, disease prevention and health promotion to maintain or improve quality of life during the life course,” might explain hepatitis B vaccine hesitancy in France. In general, higher HL is associated with greater vaccine acceptance and lower hesitancy [23]. This relationship may be influenced by the type of vaccine and the population studied. In the case of hepatitis B vaccination, controversies surrounding a potential link to demyelinating diseases [24] may contribute to differences or similarities compared to other vaccines. These specificities highlight that vaccine hesitancy is not uniform but rather a complex and multifactorial challenge to public health [25-27], frequently rooted as much in a lack of trust as in limited access to, and limited understanding of, vaccine-related information. Trust in the health care system—understood here as health agencies and medical doctors—is well-documented as a central determinant of vaccine acceptance [28,29]. However, it is often difficult to create strategies to build this trust. This gap highlights the relevance of exploring HL as a sustainable mediating lever to support trust [30]. Additionally, given that vaccine hesitancy does not necessarily regard specific vaccines but vaccination more broadly [31], any examination of opinions on a particular vaccine should also cover opinions on vaccination in general.

In this context, to enhance our understanding of hepatitis B vaccine opinions in France, this study explored the mechanisms by which HL influences the general public’s opinion on hepatitis B vaccination. We used a structural equation model [32] to investigate the direct and indirect effects of HL on these opinions. A model with 5 hypotheses was analyzed as follows: (1) a higher HL is associated with a more favorable opinion of hepatitis B vaccination (direct effect represented by the coefficient “A”); (2) a higher HL contributes positively to trust in the health care system and this trust is associated with a more favorable opinion of hepatitis B vaccination (indirect effect via “D” and “C”); (3) a higher HL has a positive influence on opinions about vaccination in general, which in turn are associated with a more favorable opinion of hepatitis B vaccination (indirect effect via “E” and “B”); (4) together, trust in the health care system and positive opinions of vaccination in general are mediators in the relationship between HL and opinions on hepatitis B vaccination (indirect effect via “D, F, and B”); and (5) the expected effect of HL on opinions of hepatitis B vaccination, in hypotheses 1 to 4, might vary according to gender, age, and financial deprivation.

## Methods

### Recruitment Procedure and Study Sample

For the present analysis, we used data from SLAVACO-Wave 3 (Suivi Longitudinal des Attitudes à l’Égard d’un Vaccin Contre la COVID-19), which was conducted between December 2 and 17, 2021. The SLAVACO project was a multiwave longitudinal survey conducted across metropolitan France. Its primary objective was to study the evolution of public attitudes toward different aspects of COVID-19 vaccination and attitudes toward vaccines more generally. Data were collected by the French Provence-Alpes-Côte d’Azur Regional Health Observatory using online self-administered questionnaires, lasting approximately 15 minutes, sent to 25,800 French adults selected by random sampling in an online panel of over 750,000 French households (Bilendi panel). The quota sampling method was then used to obtain a final sample of 2022 respondents corresponding to the adult French population in terms of gender (male and female), age (18-24, 25-34, 35-49 50-64, and 75+ years), type of employment (farmers, craftsmen, executives, intermediate professions, employees, workers, retirees, and other inactives), and population density (<2000, 2000-20,000, 20,000-100,000, and >100,000 inhabitants) in respondents’ region of residence (Alsace, Aquitaine, Auvergne, Burgundy, Brittany, Center, Île-de-France, Languedoc, Nord-Pas-de-Calais, Normandy, Pays de la Loire, and Provence-Alpes-Côte d’Azur) [33]. Final adjustments were applied by weighting the data with the raking ratio and the macro Calmar program of Statistical Analysis System. The latter was designed using census data from the French INSEE (National Institute for Statistics and Economic Studies) [34].

### Data Collected

After the participants’ consent was obtained, we assessed their opinions about vaccination in general using a question taken from the national Health Barometer survey; this survey has been conducted regularly in France over the past 2 decades [35]. The question was as follows: “Are you strongly, moderately, not really or not at all in favour of vaccination in general?” with the following 5 answer options: “yes, strongly,” “yes, moderately,” “I don’t have an opinion,” “no, not really,” and “no, not at all.” Opinions specifically on hepatitis B vaccination were also assessed in SLAVACO using a similar question as follows: “Are you strongly, moderately, not really or not at all in favour of hepatitis B vaccination?” with the same 5-option response scale. Respondents who did not answer both of these 2 questions were excluded from the present analysis.

SLAVACO also assessed HL using the European Health Literacy Survey 2019-2021 Questionnaire, HLS_19_-Q12 (12-item general health literacy questionnaire used in Health Literacy Survey 2019-2021). This questionnaire has been translated, applied, and validated in 17 countries [36,37], including France. Based on Sorensen’s matrix, the HLS_19_-Q12 comprises 12 items that measure HL in 4 cognitive dimensions (accessing, understanding, evaluating, and applying health information) and in 3 health contexts (health care, disease prevention, and health promotion) [22]. The answers to each question are selected on a 4-point scale from 0 (very difficult) to 3 (very easy). After summing the scores for all 12 items, we standardized the total score, ranging from 0 to 100, where a higher score reflected a higher level of HL. Internal consistency was excellent with a Cronbach α coefficient of 0.91.

To measure trust in the health care system, we used the methodology used in the 2021 Political Confidence Barometer, the main French longitudinal study on public trust in politics [38]. We measured 2 dimensions: trust in government health agencies and trust in medical doctors. A 5-point scale ranging from 0 (no trust at all) to 4 (complete trust) was used for each measure.

Our analysis also included self-reported socioeconomic variables. Specifically, gender was reported using 3 categories (man, woman, and other), and age was categorized into the following groups: 18-24, 25-34, 35-49, 50-64, 65-74, and 75+ years, in line with the INSEE’s age categories [39]. Educational attainment was measured using 6 categories: no educational qualification, lower secondary school certificate, upper secondary school certificate, bachelor’s degree, master’s degree, and doctorate, while employment status was recorded as currently employed, not employed, or retired. Financial deprivation was determined using the question “How easy or difficult is it for you to pay all your bills at the end of the month?” Respondents who answered “difficult” or “very difficult” were classified as having financial difficulties. Finally, the presence of one or more chronic diseases was assessed using a “yes” or ”no” question.

### Statistical Analyses

Continuous variables were reported in terms of the mean and SD, while categorical variables were reported in terms of frequency and percentages. We performed descriptive analyses to explore covariables associated with opinions on hepatitis B vaccination. To do this, we treated these opinions and opinions on vaccination in general as binary variables: a favorable opinion was defined as an answer of “yes, strongly” or “yes, moderately.” An unfavorable opinion was defined as responding “I don’t have an opinion,” “no, not really,” or “no, not at all.” For the bivariate and mediation analyses, a 0 to 4 scale was used (“no, not at all”=0; “no, not really”=1; “I don’t have an opinion”=2; “yes, moderately”=3; and “yes, strongly”=4).

We conducted a Pearson correlation analysis to investigate the bivariate association between the 5 variables of interest (ie, opinions on vaccination in general, opinions on hepatitis B vaccination, HL level, trust in government health agencies, and trust in medical doctors).

**Figure 1:**
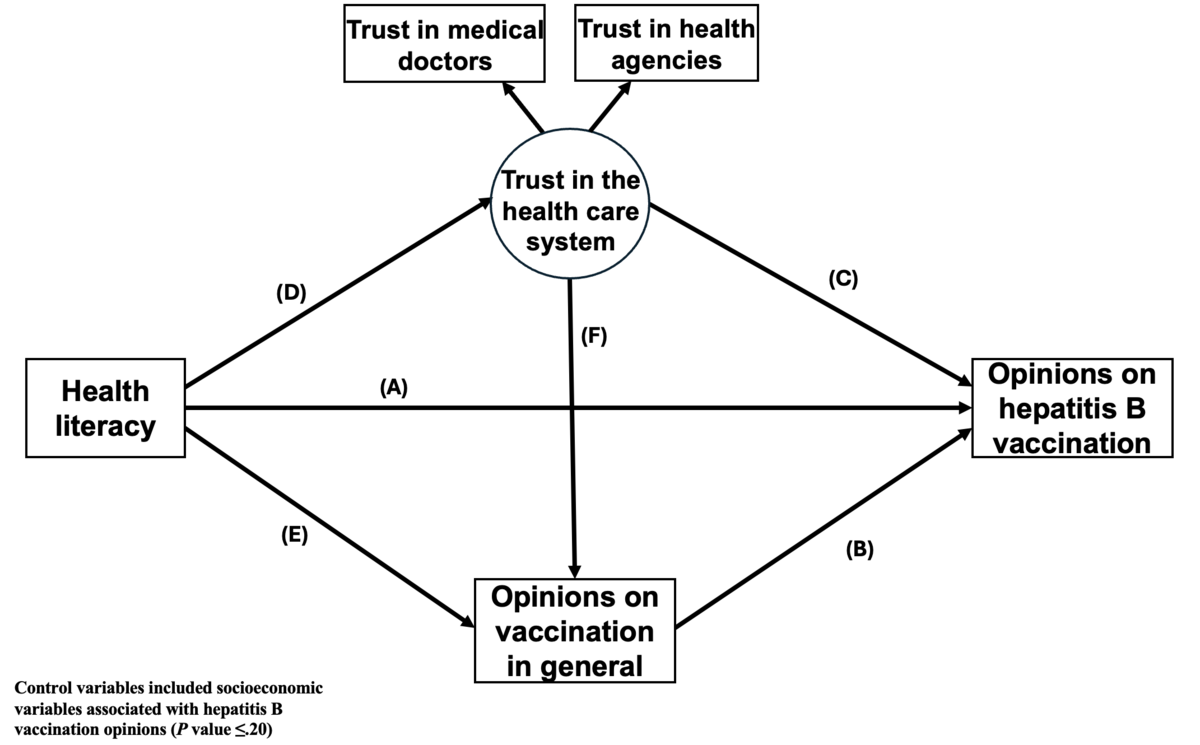
Hypothesized mediation model for health literacy’s effect on hepatitis B vaccination opinions (A-F are the coefficients from the Introduction).

We performed a mediation analysis to explore whether favorable opinions toward vaccination in general and trust in the health care system mediated the association between HL and favorable opinions toward hepatitis B vaccination (Figure 1). Trust in the health care system was modelled using a latent variable based on 2 observed indicators: trust in government health agencies and trust in medical doctors. Possible mediation was then examined after stratification by gender (man vs woman), age (<50 years vs ≥50 years), and financial deprivation (yes vs no). The control variables included socioeconomic variables associated with hepatitis B vaccination opinions (*P* value ≤.20). The 95% CI for the direct, indirect, and total effects of the mediation models were estimated using 5000 bootstrap samples. The effects of HL were estimated per 10-point increase in the HL score. We used a series of indicators to assess the goodness-of-fit of the model: Tucker-Lewis Index (considered excellent above 0.95); comparative fit index (considered excellent above 0.95); and root-mean-square error of approximation (RMSEA, considered excellent if below 0.05).

All analyses were performed on the weighted database. Analyses were carried out with statistical R software (version 4.4.1; R Foundation), and the significance level was set to 5% for all 2-sided tests.

### Ethical Considerations

This study was approved in France by the Ethics Evaluation Committee of the National Institute of Health and Medical Research (IRB00003888) on February 9, 2021 (21-770). After receiving information about SLAVACO-Wave 3, informed consent was obtained from all the participants before they started the survey. In accordance with standards practice for web-based surveys, we did not have access to any data that could identify respondents. Participants received no compensation.

## Results

### Sample Characteristics

Among the 2022 individuals who participated in SLAVACO-Wave 3, we excluded 90 (4.4%) because of missing answers to the questions on opinions on hepatitis B vaccination and/or vaccination in general. Of the 1932 adults comprising this study’s sample, 52.6% (n=1016) were women and 34.1% (n=658) were retirees (Table 1). Most of the participants (n=1184, 61.4%) had at least an upper secondary school certificate, and most (n=1210, 62.6%) did not report financial difficulties. Half of the participants (n=952, 49.3%) reported one or more chronic diseases. A quarter (n=495, 25.6%) expressed an unfavorable opinion about hepatitis B vaccination, and a fifth (n=375, 19.4%) towards vaccination in general. Among those hesitant toward hepatitis B vaccination, 58.6% (290/495) were nevertheless favorable to vaccination in general, while 18.6% (290/1557) of those favorable to vaccination in general expressed hesitancy toward hepatitis B vaccination.

**Table 1:** Description of this study’s sample and factors associated with opinions on hepatitis B vaccination in France (N=1932).

Variables			Overall (N=1932)		Opinions on hepatitis B vaccination				*P* value
					Favorable (n=1437, 74.4%)		Unfavorable (n=495, 25.6%)		
**Age group (years), n (%)**									.14
	18-24	201 (10.4)		157 (78.4)		43 (21.6)			
	25-34	282 (14.6)		216 (76.5)		66 (23.5)			
	35-49	460 (23.8)		345 (75)		115 (25)			
	50-64	466 (24.1)		324 (69.5)		142 (30.5)			
	65-74	279 (14.4)		211 (75.8)		68 (24.2)			
	75+	244 (12.6)		183 (75.1)		61 (24.9)			
**Gender, n (%)**									<.001
	Man	916 (47.4)		739 (80.7)		177 (19.3)			
	Woman	1016 (52.6)		698 (68.7)		318 (31.3)			
**Employment status, n (%)**									.30
	Currently employed	802 (41.5)		611 (76.1)		191 (23.9)			
	Unemployed	472 (24.4)		339 (71.9)		133 (28.1)			
	Retired	658 (34.1)		487 (74)		171 (26)			
**Educational attainment, n (%)**									.01
	No diploma	121 (6.3)		88 (72.7)		33 (27.3)			
	Lower secondary school certificate	626 (32.4)		452 (72.3)		174 (27.7)			
	Upper secondary school certificate	405 (21)		283 (69.8)		123 (30.2)			
	Bachelor’s degree	347 (18)		266 (76.6)		81 (23.4)			
	Master’s degree	208 (10.8)		171 (82)		37 (18)			
	Doctorate	224 (11.6)		177 (78.8)		48 (21.2)			
**Financial deprivation, n (%)**									<.001
	Yes	722 (37.4)		503 (69.7)		219 (30.3)			
	No	1210 (62.6)		934 (77.2)		276 (22.8)			
**Chronic diseases, n (%)**									.02
	Yes	952 (49.3)		730 (76.7)		202 (23.3)			
	No	980 (50.7)		707 (72.1)		274 (27.9)			
Health literacy score (continuous), mean (SD)			61.1 (17.0)		62.6 (16.9)		57.0 (16.8)		<.001
**Opinion on vaccination in general, n (%)**									<.001
	Favorable	1557 (80.6)		1267 (81.4)		290 (18.6)			
	Unfavorable	375 (19.4)		170 (45.3)		205 (54.7)			
“**Do you trust government health agencies?” n (%)**									<.001
	Not at all (0)	358 (18.5)		197 (54.9)		161 (45.1)			
	Not really (1)	571 (29.5)		414 (72.5)		157 (27.5)			
	No opinion (2)	80 (4.1)		46 (57.9)		33 (42.1)			
	Moderately (3)	805 (41.7)		678 (84.2)		127 (15.8)			
	Completely (4)	118 (6.1)		102 (86.3)		16 (13.7)			
“**Do you trust medical doctors?” n (%)**									<.001
	Not at all (0)	50 (2.6)		30 (60.6)		20 (39.4)			
	Not really (1)	148 (7.6)		90 (60.6)		58 (39.4)			
	No opinion (2)	40 (2.1)		22 (55.4)		18 (44.6)			
	Moderately (3)	1232 (63.8)		901 (73.1)		331 (26.9)			
	Completely (4)	462 (23.9)		393 (85.2)		69 (14.8)			

Approximately half the sample (n=923, 47.8%) reported trusting government health agencies, while 87.7% (n=1694) reported trusting medical doctors. The mean HL score in the sample was 61.1 (SD 17.0). Individuals with a favorable opinion of hepatitis B vaccination had a higher HL score than those with an unfavorable opinion (62.6 vs 57.0, *P*<.001).

### Correlation Analyses

The correlation matrix for all variables in the mediation model is reported in Table 2. A favorable opinion on hepatitis B vaccination was associated with a higher HL level (*r*=0.18, *P*<.001), a favorable opinion on vaccination in general (*r*=0.44, *P*<.001), a higher level of trust in government health agencies (*r*=0.27, *P*<.001), and a higher level of trust in medical doctors (*r*=0.22, *P*<.001). A higher HL level was positively and significantly correlated with a positive opinion on vaccination in general (*r*=0.20, *P*<.001), with a higher level of trust in government health agencies (*r*=0.27, *P*<.001), and with a higher level of trust in medical doctors (*r*=0.27, *P*<.001). A favorable opinion on vaccination in general was positively associated with a higher level of trust in government health agencies (*r*=0.27, *P*<.001), and with a higher level of trust in medical doctors (*r*=0.27, *P*<.001).

**Table 2:** Correlation matrix of mediation model variables.

Variables		Health literacy (A)	Opinion on hepatitis B vaccination (B)	Opinion on vaccination in general (C)	Trust in government health agencies (D)	Trust in medical doctors (E)
**Health literacy (A)**						
	*r*	1	0.18	0.20	0.27	0.27
	*P* value	—^a^	<.001	<.001	<.001	<.001
**Opinion on hepatitis B vaccination (B)**						
	*r*	0.18	1	0.44	0.27	0.22
	*P* value	<.001	—	<.001	<.001	<.001
**Opinion on vaccination in general (C)**						
	*r*	0.20	0.44	1	0.27	0.27
	*P* value	<.001	<.001	—	<.001	<.001
**Trust in government health agencies (D)**						
	*r*	0.27	0.27	0.27	1	0.34
	*P* value	<.001	<.001	<.001	—	<.001
**Trust in medical doctors (E)**						
	*r*	0.27	0.22	0.27	0.34	1
	*P* value	<.001	<.001	<.001	<.001	—

^a^Not available.

### Mediation Analyses

**Figure 2:**
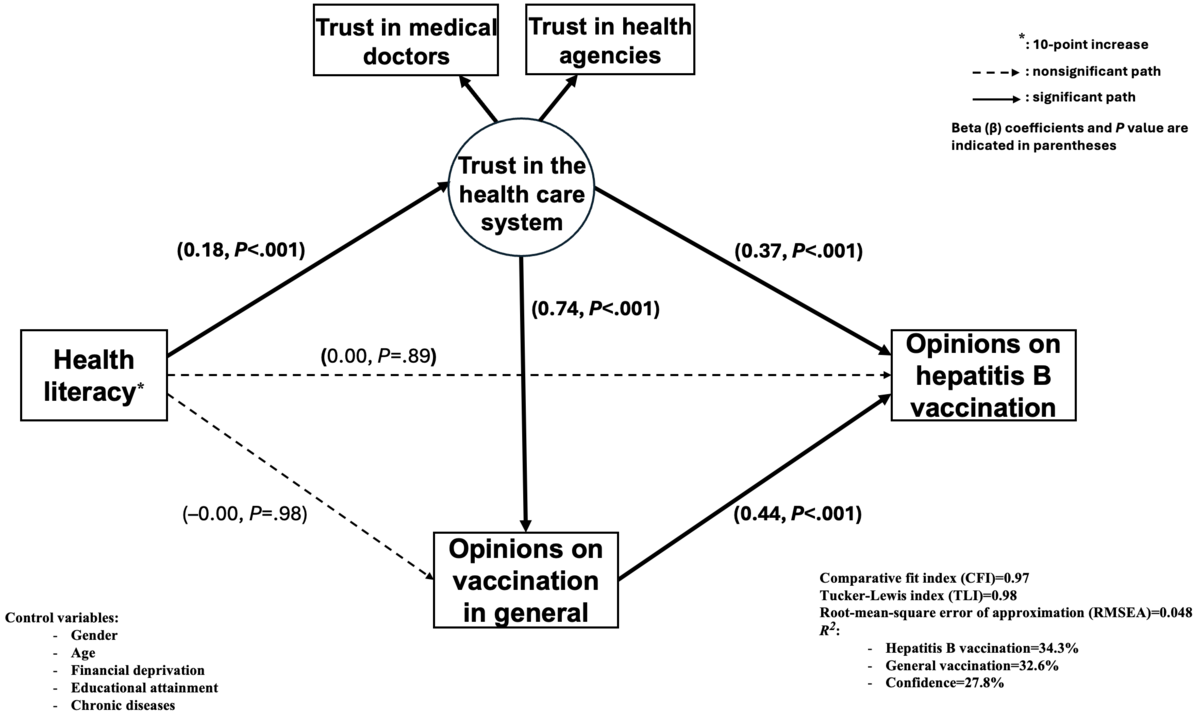
Mediation model of health literacy on opinions about hepatitis B vaccination (N=1932).

The mediation model for all participants, with 2 mediators and a direct path, is presented in Figure 2. A significant and positive relationship was observed between trust in the health care system and a favorable hepatitis B vaccination opinion (β=0.37; *P*<.001). There was also a significant positive relationship between a favorable opinion on vaccination in general and a favorable opinion on hepatitis B vaccination (β=0.44; *P*<.001). A higher level of trust in the health care system had a significant and strong positive association with a favorable opinion on vaccination in general (β=0.74; *P*<.001). A higher HL level was positively and significantly associated with a higher level of trust in the health care system (β=0.18; *P*<.001), but not associated with a favorable opinion on vaccination in general and with a favorable opinion on hepatitis B vaccination (β=0.00; *P*=.89 and β=–0.00; *P*=.98, respectively). All estimations were adjusted for educational attainment and the presence of chronic diseases.

**Figure 3:**
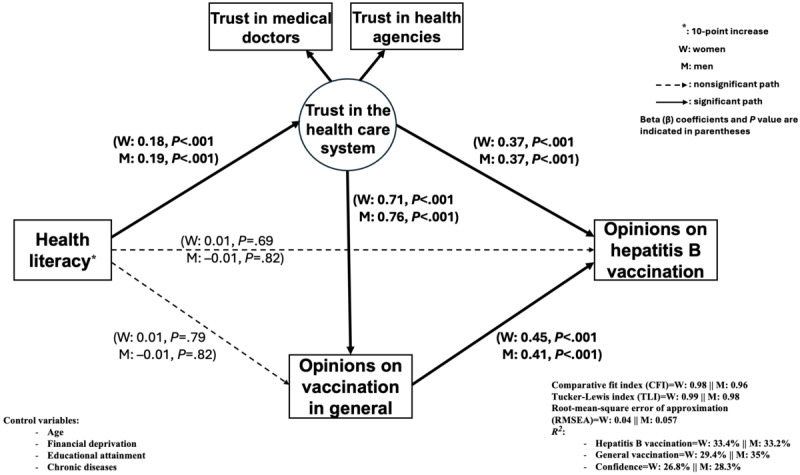
Mediation model of health literacy on opinions about hepatitis B vaccination according to gender (number of women=1016; number of men=916).

After stratifying by gender (Figure 3), we found that a higher level of trust in the health care system was positively associated with a favorable opinion on hepatitis B vaccination in women and men (β=0.37; *P*<.001 for both). A higher level of trust in the health care system was also significantly associated with a favorable opinion about vaccination in general for both genders (women: β=0.71; *P*<.001 and men: β=0.76; *P*<.001). A higher HL level was positively associated with a higher level of trust in the health care system for both genders (women: β=0.18; *P*<.001 and men: β=0.19; *P*<.001) but had no direct effect on a favorable opinion about vaccination in general or about hepatitis B vaccination.

**Figure 4:**
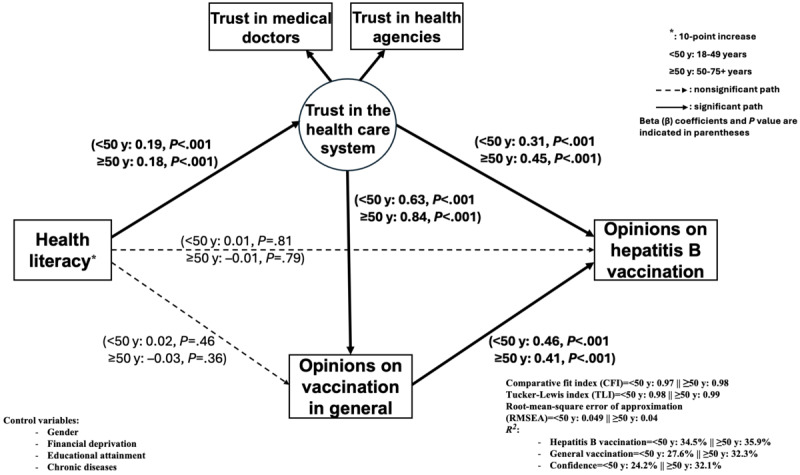
Mediation model of health literacy on opinions about hepatitis B vaccination according to age (number of participants aged <50 years=943; number of participants aged ≥50 years=989).

In terms of age (Figure 4), similar associations were observed in the <50 years and ≥50 years age groups. A higher level of trust was positively associated with a favorable opinion on hepatitis B vaccination, with a stronger association observed in adults ≥50 years (β=0.45; *P*<.001) than in younger adults (β=0.31; *P*<.001). The association between trust in the health care system and a favorable opinion on vaccination in general was strong for both age groups (<50 years: β=0.63; ≥50 years: β=0.84; *P*<.001). A higher HL level had a positive influence on trust in the health care system in both age groups (<50 years: β=0.19; ≥50 years: β=0.18; *P*<.001) but had no significant direct effect on a favorable opinion about vaccination (general or hepatitis B).

**Figure 5:**
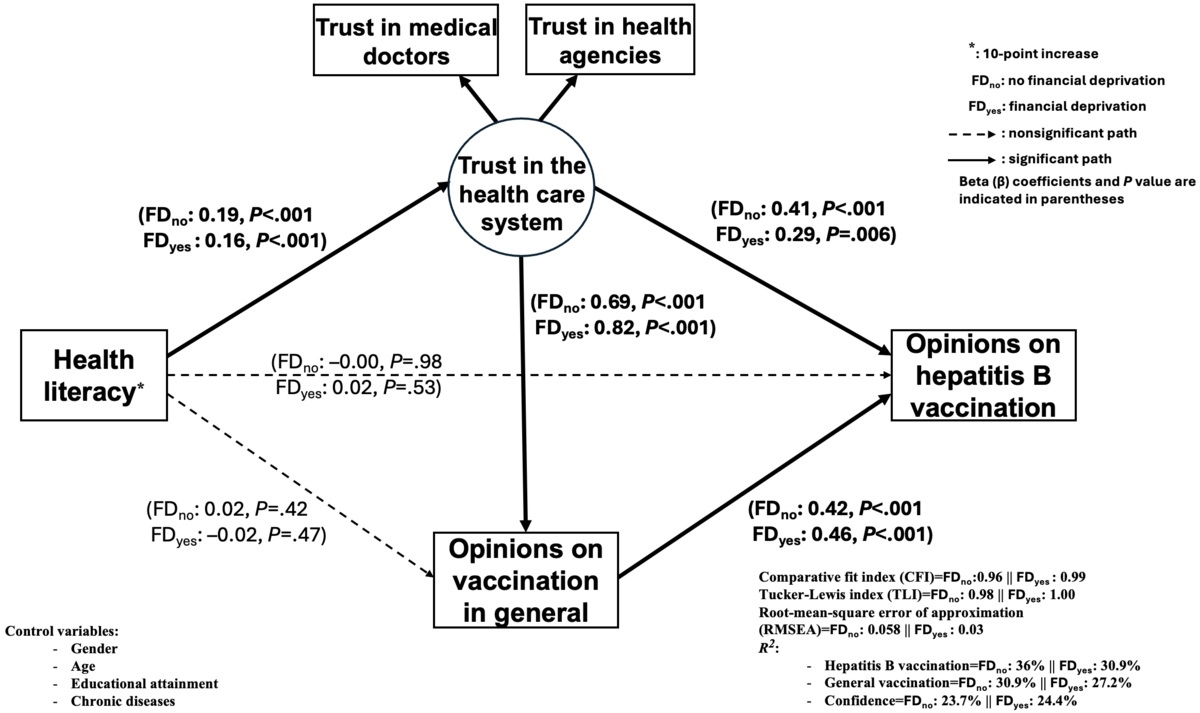
Mediation model of health literacy on opinions about hepatitis B vaccination according to financial deprivation (number of participants with no financial deprivation=1210; number of participants with financial deprivation=722).

Lastly, after stratifying by financial deprivation (Figure 5), the association between trust in the health care system and a favorable hepatitis B vaccination opinion was stronger among individuals who did not have financial deprivation (β=0.41; *P*<.001) than among those who did (β=0.29; *P*=.006). In contrast, the association between trust in the health care system and a favorable opinion on vaccination in general was more pronounced among individuals who had financial difficulties (β=0.82; *P*<.001) than among those who did not (β=0.69; *P*<.001). A higher HL level remained positively associated with trust in the health care system in both subgroups (no financial deprivation: β=0.19; financial deprivation: β=0.16; *P*<.001), although there was no direct effect on favorable vaccination opinions (in general or hepatitis B).

Table 3 shows that the effect of HL on hepatitis B vaccination opinions among all participants was fully mediated by trust in the health care system, after adjusting for educational attainment and the presence of chronic diseases. When trust was the only mediator, the indirect effect of HL was estimated at 0.068 (95% CI 0.042-0.093), accounting for 52.4% (0.068/0.1297) of the total effect. When trust and opinion on vaccination in general were combined as parallel mediators, the indirect effect attributable to HL was 0.059 (95% CI 0.048-0.07), representing 45.5% (0.059/0.1297) of the total effect. Mediation analyses stratified by gender, age, and financial deprivation status also confirmed the complete mediation of the relationship between HL and hepatitis B vaccination opinion through trust in the health care system, even after adjustment for different sets of control variables depending on the stratum (gender, age, educational attainment, financial deprivation, and chronic diseases). The total effect of HL on hepatitis B vaccination opinion was more pronounced among women and those without financial deprivation.

**Table 3:** Direct, indirect, and total effects for the mediation model of health literacy on opinions about hepatitis B vaccination (nonstratified model and models stratified by gender, age, and financial deprivation).

Model pathways			Estimated effects		95% CIs		*P* value
**Direct effect (H1; health literacy^a^>hepatitis B vaccination)**							
	All participants^b^ (N=1932)	0.003		–0.035 to 0.04		.89	
	Women^c^ (n=1016)	0.01		–0.04 to 0.06		.69	
	Men^c^ (n=916)	–0.006		–0.062 to 0.05		.82	
	18-49 years^d^ (n=943)	0.005		–0.036 to 0.046		.81	
	50-75+ years^d^ (n=989)	–0.006		–0.051 to 0.039		.79	
	No financial deprivation^e^ (n=1210)	–0.001		–0.05 to 0.048		.98	
	Financial deprivation^e^ (n=722)	0.015		–0.033 to 0.063		.53	
**Indirect effects (H2; health literacy^a^>vaccination in general>hepatitis B vaccination)**							
	All participants^b^ (N=1932)	–0		–0.017 to 0.017		.98	
	Women^c^ (n=1016)	0.003		–0.020 to 0.027		.79	
	Men^c^ (n=916)	–0.003		–0.028 to 0.022		.81	
	18-49 years^d^ (n=943)	0.009		–0.015 to 0.032		.47	
	50-75+ years^d^ (n=989)	–0.01		–0.032 to 0.011		.34	
	No financial deprivation^e^ (n=1210)	0.009		–0.013 to 0.031		.43	
	Financial deprivation^e^ (n=722)	–0.01		–0.035 to 0.016		.46	
**Indirect effects (H3; health literacy^a^> trust>hepatitis B vaccination)**							
	All participants^b^ (N=1932)	0.068		0.042 to 0.093		<.001	
	Women^c^ (n=1016)	0.066		0.033 to 0.100		<.001	
	Men^c^ (n=916)	0.071		0.033 to 0.11		<.001	
	18-49 years^d^ (n=943)	0.059		0.031 to 0.087		<.001	
	50-75+ years^d^ (n=989)	0.084		0.042 to 0.126		<.001	
	No financial deprivation^e^ (n=1210)	0.079		0.046 to 0.112		<.001	
	Financial deprivation^e^ (n=722)	0.048		0.012 to 0.083		.008	
**Indirect effects (H4; health literacy^a^>trust>vaccination in general>hepatitis B vaccination)**							
	All participants^b^ (N=1932)	0.059		0.048 to 0.070		<.001	
	Women^c^ (n=1016)	0.058		0.044 to 0.073		<.001	
	Men^c^ (n=916)	0.06		0.044 to 0.077		<.001	
	18-49 years^d^ (n=943)	0.056		0.041 to 0.07		<.001	
	50-75+ years^d^ (n=989)	0.063		0.046 to 0.08		<.001	
	No financial deprivation^e^ (n=1210)	0.056		0.043 to 0.069		<.001	
	Financial deprivation^e^ (n=722)	0.062		0.044 to 0.083		<.001	
**Total effect**							
	All participants^b^ (N=1932)	0.129		0.101 to 0.157		<.001	
	Women^c^ (n=1016)	0.138		0.101 to 0.177		<.001	
	Men^c^ (n=916)	0.123		0.08 to 0.165		<.001	
	18-49 years^d^ (n=943)	0.128		0.087 to 0.169		<.001	
	50-75+ years^d^ (n=989)	0.13		0.092 to 0.168		<.001	
	No financial deprivation^e^ (n=1210)	0.143		0.106 to 0.181		<.001	
	Financial deprivation^e^ (n=722)	0.115		0.073 to 0.158		<.001	

^a^10-point increase.

^b^Estimated effects are controlled for gender, age, educational attainment, financial deprivation, and chronic diseases.

^c^Estimated effects are controlled for age, educational attainment, financial deprivation, and chronic diseases.

^d^Estimated effects are controlled for gender, educational attainment, financial deprivation, and chronic diseases.

^e^Estimated effects are controlled for gender, age, educational attainment, and chronic diseases.

For the control variables (Multimedia Appendix 1), the presence of chronic diseases was significantly associated with a higher level of trust in the health care system (β=0.11; *P*=.004), particularly among women (β=0.15; *P*=.004) and individuals without financial deprivation (β=0.14; *P*=.006). About educational attainment, having only a lower or upper secondary school certificate was negatively associated with the level of trust in the health care system among women (lower secondary school certificate β=–0.29; *P*=.004; upper secondary school certificate β=–0.23; *P*=.03) and among financially deprived individuals (β=–0.27; *P*=.01 and β=–0.26; *P*=.03, respectively).

All models showed an excellent goodness-of-fit (Tucker-Lewis Index >0.95, comparative fit index >0.95, and RMSEA <0.05), except for the men stratum (RMSEA=0.057) and for individuals without financial deprivation (RMSEA=0.058).

## Discussion

### Principal Findings

France has one of the highest rates of vaccine hesitancy in the world. According to an international study conducted in 2015, a total of 41% of French people considered that vaccines might be dangerous, which was the highest rate among the 67 countries studied [40]. The significant proportion of individuals expressing an unfavorable opinion on hepatitis B vaccination (495/1932, 25.6%) in our present study highlights the persisting skepticism concerning vaccines in the French population. Our findings highlight that this skepticism was more pronounced than for vaccination in general. The moderate correlation between opinions on vaccination in general and those on hepatitis B vaccination (*r*=0.44) indicates that these 2 dimensions only partially overlap; this justifies the detailed focus on this specific vaccine.

The particularly high level of skepticism toward hepatitis B vaccination, which we identified, could be the result of a historical context marked by health controversies, particularly in the 1990s, when cases of central nervous system demyelination after hepatitis B vaccination raised fears in the general public of a potential link [41]. Although subsequent investigations did not establish a causal link [24,42] and even though French authorities officially declared that there was no risk of developing a demyelinating disease from hepatitis B vaccination [43], the impact of these events on the general perception of hepatitis B vaccination persists today.

Our study results showed that women, people with a lower level of educational attainment, and individuals with no chronic disease were all significantly more likely to have an unfavorable opinion about hepatitis B vaccination. This greater vaccine hesitancy in women might reflect their greater engagement with health information than men. This engagement may, in turn, leave them more exposed to negative discourses, especially if they echo personal or shared experiences linked to past health controversies [44]. Although men in our study were more likely to have a favorable opinion of hepatitis B vaccination, some studies have suggested that women are more invested in health care and child-rearing than men, and so are more likely to get this vaccine [45,46].

The significant association that we found between the presence of chronic diseases and a higher level of trust in the health care system might partly explain the more favorable opinions toward hepatitis B vaccination, which we observed among people with chronic diseases. In addition to a lack of trust in the health care system, the greater reluctance to hepatitis B vaccination we observed in individuals with no chronic disease could be explained by greater complacency, identified as one of the key determinants of vaccine hesitancy. Complacency occurs when the perception of risk from vaccine-preventable diseases is low and when vaccination is not perceived as necessary [47]. Without a perceived risk to their health and without regular contact with health care services, these individuals might develop a less favorable opinion about vaccination.

The association we observed between intermediate educational attainment (ie, having only a lower or upper secondary school certificate) and less trust in the health care system among women and people in financial difficulty suggests that this level of education may sometimes be associated with a more skeptical attitude toward medical recommendations. This association is also reflected in the relatively high proportion of individuals in our study who expressed an unfavorable opinion about hepatitis B vaccination within these 2 subgroups. About the influence of educational attainment on vaccination opinions, findings in the literature are mixed; some studies emphasize its central role in shaping provaccination opinions [40,48], while others show that individuals with higher educational attainment might also be more likely to adopt skeptical attitudes [49]. These contrasting views highlight the need to develop other, more actionable and multidimensional measures, such as HL.

In our study, a favorable hepatitis B vaccination opinion was significantly positively associated with a higher HL level before mediation was taken into account. In the mediation model, this association was fully mediated by trust in the health care system, even after stratifying by gender, age group, and financial difficulties. There was no significant direct relationship between HL and opinions on vaccination (ie, general or hepatitis B) when trust was considered in all strata. Although no study to date has directly investigated the mediating role of trust in the health care system on the relationship between HL and hepatitis B vaccination opinion, many studies have highlighted the positive influence of HL and trust in the health care system on hepatitis B prevention behaviors. For example, a group study of sociocultural barriers to hepatitis B prevention among Korean Americans suggested that there were specific HL barriers that governed individuals’ behavior in terms of hepatitis B prevention and care access [50]. Similarly, in a study in the Democratic Republic of Congo [51], trust in the health care staff increased the public’s acceptance and the effectiveness of community initiatives to prevent mother-to-child transmission of the disease, especially initiatives focusing on adherence to vaccination and antiviral uptake. HL could become an essential tool to overcome the challenge of improving trust in the health care system to reduce vaccine hesitancy [52]. By strengthening individuals’ ability to understand how the health care system functions, to evaluate the reliability of health information, and to communicate effectively with professionals in order to both avoid negative interactions and engage proactively in their own care [53], HL could contribute to creating sustainable trust in the health care system.

Our key finding—the absence of a direct relationship between HL and a favorable opinion on hepatitis B vaccination—might be specific to this vaccination. This is because, in order to have a favorable opinion, a degree of trust in the expected long-term benefits (which are not visible) is needed, especially given the controversy surrounding this vaccine in France. This finding may also reflect the specific nature of vaccination as a public health issue; vaccination is particularly exposed to conflicting information at the heart of political and social debate, and is increasingly shaped by dynamics of trust or the lack thereof, which in turn are often associated with collective reasoning. Accordingly, the absence of a direct relationship between HL and opinions on vaccination in general, which we observed in the mediation model, might indicate the limitations of using an approach based solely on increasing general HL to combat vaccine hesitancy. This is a very important point, because over the last decade, the vaccine debate has become a way for the French public to express social tensions and political mistrust [54]. As our results suggest, interventions to improve the level of HL, such as cross-cutting strategies, might contribute to strengthening trust in the health care system.

### Limitations

This study has limitations. First, its cross-sectional design prevented us from establishing causal relationships between the variables analyzed. Second, the quota sampling method we used does not guarantee the geographical and socioeconomic representativeness of the whole population of French adults. Our findings must therefore be interpreted with caution. Third, the SLAVACO survey was conducted during the COVID-19 pandemic, when vaccination was the subject of intense media attention, frequently affected by misinformation and disinformation. This may have strongly influenced participants’ opinions on vaccination and amplified the mediating effect of trust in the health care system, which we observed. Lastly, following the framework by Parker [55], HL is influenced by both internal (cognitive, educational, and socioeconomic) and external (social, health care, and macroeconomic) factors. Our study did not systematically assess these determinants, highlighting the need for tools that capture the full spectrum of HL in future research.

### Conclusions

Enhancing the level of HL may lead to greater trust in government health agencies and medical doctors, which are 2 essential dimensions for positively influencing hepatitis B vaccination opinions. To achieve the WHO’s goal of hepatitis elimination by 2030, future public health strategies in France should take HL and these 2 dimensions into account to effectively reduce the country’s currently high rate of hepatitis B vaccine hesitancy. From a public health perspective, these findings suggest that strategies should not only focus on providing clear information about vaccines and ensuring access, but also on actively strengthening trust in health care institutions and professionals. National communication campaigns aimed at correcting misconceptions about hepatitis B vaccination could be complemented by targeted interventions for groups most likely to hold negative opinions, such as women, individuals with intermediate education levels, those without chronic diseases, and higher-income populations. In parallel, incorporating HL modules into school curricula and community programs could provide a sustainable means of fostering trust and supporting informed decision-making.

For future research, it will be important to develop and apply vaccine-specific HL tools, to longitudinally assess the causal relationships between HL and the key determinants of vaccine hesitancy (7C-model), and to compare these dynamics across different vaccines. A broader assessment of trust encompassing all 6 components of the health care system—governance, financing, service delivery, human resources, health products and interventions, and health information—as defined by the WHO [56], would also provide a more comprehensive understanding. Conducting a similar survey in the post–COVID-19 context could also reveal different trends, reflecting the evolving public perceptions of vaccines and health authorities.

## Appendix

Multimedia Appendix 1 Table showing estimates of control variables for different mediation models.

## Abbreviations

HBV: hepatitis B virus
HL: health literacy
HLS19-Q12: 12-item general health literacy questionnaire used in Health Literacy Survey 2019-2021
INSEE: National Institute for Statistics and Economic Studies
RMSEA: root-mean-square error of approximation
SLAVACO: Suivi Longitudinal des Attitudes à l’Égard d’un Vaccin Contre la COVID-19
WHO: World Health Organization

## Data Availability

The datasets used and/or analyzed during this study are available from the corresponding author on reasonable request. Information and agreement to use HLS_19_-Q12 (12-item general health literacy scale) can be found on the M-POHL (Action Network on Measuring Population and Organizational Health Literacy) web page.
