# Supplementary material for: Mediation Analysis of the Relationship Between Health Literacy and the French General Population’s Opinions on Hepatitis B Vaccination: Representative Cross-Sectional Survey of the SLAVACO Project
Source: JMIR Public Health Surveill. 2026 Feb 13;12:e82496. doi: 10.2196/82496 (PMC12949401; doi:10.2196/82496)
Supplement: Multimedia Appendix 1 [file publichealth_v12i1e82496_app1.docx]

- **All participants**

| **Model pathways** | **Estimated** | ***P*-value** |
| --- | --- | --- |
| *(Gender 🡪 Opinions on hepatitis B vaccination)* |  |  |
| Men | 1 |  |
| Women | -0.18 | **.002** |
| *(Age 🡪 Opinions on hepatitis B vaccination)* |  |  |
| 50-75+ | 1 |  |
| 18-49 | 0.27 | **<.001** |
| *(Financial deprivation 🡪 Opinions on hepatitis B vaccination)* |  |  |
| No | 1 |  |
| Yes | 0.07 | .31 |
| *(One or more chronic disease 🡪 Opinions on hepatitis B vaccination)* |  |  |
| No | 1 |  |
| Yes | 0.09 | .13 |
| *(Education level 🡪 Opinions on hepatitis B vaccination)* |  |  |
| No diploma | 1 |  |
| Lower secondary school certificate | -0.02 | .89 |
| Upper secondary school certificate | -0.15 | .28 |
| Bachelor’s degree | -0.06 | .66 |
| Master’s degree | 0.03 | .84 |
| Doctorate degree | -0.03 | .83 |
| *(Gender 🡪 Opinions on vaccination in general)* |  |  |
| Men | 1 |  |
| Women | -0.13 | **.03** |
| *(Age 🡪 Opinions on vaccination in general)* |  |  |
| 50-75+ | 1 |  |
| 18-49 | -0.33 | **<.001** |
| *(Financial deprivation 🡪 Opinions on vaccination in general)* |  |  |
| No | 1 |  |
| Yes | -0.16 | **.02** |
| *(One or more chronic disease 🡪 Opinion on vaccination in general)* |  |  |
| No | 1 |  |
| Yes | 0.08 | .18 |
| *(Education level 🡪 Opinion on vaccination in general)* |  |  |
| No diploma | 1 |  |
| Lower secondary school certificate | 0.15 | .23 |
| Upper secondary school certificate | 0.22 | .09 |
| Bachelor’s degree | 0.13 | .32 |
| Master’s degree | 0.25 | .09 |
| Doctorate degree | 0.50 | **.001** |
| *(Gender 🡪 Trust in the healthcare system)* |  |  |
| Men | 1 |  |
| Women | -0.14 | **<.001** |
| *(Age 🡪 Trust in the healthcare system)* |  |  |
| 50-75+ | 1 |  |
| 18-49 | -0.13 | **.001** |
| *(Financial deprivation 🡪 Trust in the healthcare system)* |  |  |
| No | 1 |  |
| Yes | -0.15 | **<.001** |
| *(One or more chronic disease 🡪 Trust in the healthcare system)* |  |  |
| No | 1 |  |
| Yes | 0.11 | **.004** |
| *(Education level 🡪 Trust in the healthcare system)* |  |  |
| No diploma | 1 |  |
| Lower secondary school certificate | -0.14 | **.049** |
| Upper secondary school certificate | -0.09 | .21 |
| Bachelor’s degree | -0.01 | .93 |
| Master’s degree | 0.10 | .25 |
| Doctorate degree | 0.08 | .36 |

- **Women**

| **Model pathways** | **Estimated** | **p-value** |
| --- | --- | --- |
| *(Age 🡪 Opinions on hepatitis B vaccination)* |  |  |
| 50-75+ | 1 |  |
| 18-49 | 0.32 | **<.001** |
| *(Financial deprivation 🡪 Opinions on hepatitis B vaccination)* |  |  |
| No | 1 |  |
| Yes | 0.05 | .57 |
| *(One or more chronic disease 🡪 Opinions on hepatitis B vaccination)* |  |  |
| No | 1 |  |
| Yes | -0.01 | .93 |
| *(Education level 🡪 Opinions on hepatitis B vaccination)* |  |  |
| No diploma | 1 |  |
| Lower secondary school certificate | -0.01 | .93 |
| Upper secondary school certificate | -0.12 | .94 |
| Bachelor’s degree | -0.02 | .92 |
| Master’s degree | -0.06 | .78 |
| Doctorate degree | 0.00 | .99 |
| *(Age 🡪 Opinions on vaccination in general)* |  |  |
| 50-75+ | 1 |  |
| 18-49 | -0.30 | **<.001** |
| *(Financial deprivation 🡪 Opinions on vaccination in general)* |  |  |
| No | 1 |  |
| Yes | -0.25 | **.004** |
| *(One or more chronic disease 🡪 Opinion on vaccination in general)* |  |  |
| No | 1 |  |
| Yes | 0.09 | .29 |
| *(Education level 🡪 Opinion on vaccination in general)* |  |  |
| No diploma | 1 |  |
| Lower secondary school certificate | 0.16 | .36 |
| Upper secondary school certificate | 0.22 | .21 |
| Bachelor’s degree | 0.10 | .58 |
| Master’s degree | 0.26 | .19 |
| Doctorate degree | 0.35 | .10 |
| *(Age 🡪 Trust in the healthcare system)* |  |  |
| 50-75+ | 1 |  |
| 18-49 | -0.10 | .06 |
| *(Financial deprivation 🡪 Trust in the healthcare system)* |  |  |
| No | 1 |  |
| Yes | -0.13 | **.01** |
| *(One or more chronic disease 🡪 Trust in the healthcare system)* |  |  |
| No | 1 |  |
| Yes | 0.15 | **.004** |
| *(Education level 🡪 Trust in the healthcare system)* |  |  |
| No diploma | 1 |  |
| Lower secondary school certificate | -0.29 | **.004** |
| Upper secondary school certificate | -0.23 | **.03** |
| Bachelor’s degree | -0.12 | .27 |
| Master’s degree | -0.01 | .92 |
| Doctorate degree | -0.09 | .49 |

- **Men**

| **Model pathways** | **Estimated** | **p-value** |
| --- | --- | --- |
| *(Age 🡪 Opinions on hepatitis B vaccination)* |  |  |
| 50-75+ | 1 |  |
| 18-49 | 0.22 | **.02** |
| *(Financial deprivation 🡪 Opinions on hepatitis B vaccination)* |  |  |
| No | 1 |  |
| Yes | 0.10 | .32 |
| *(One or more chronic disease 🡪 Opinions on hepatitis B vaccination)* |  |  |
| No | 1 |  |
| Yes | 0.21 | **.02** |
| *(Education level 🡪 Opinions on hepatitis B vaccination)* |  |  |
| No diploma | 1 |  |
| Lower secondary school certificate | 0.01 | .97 |
| Upper secondary school certificate | -0.14 | .50 |
| Bachelor’s degree | -0.08 | .70 |
| Master’s degree | 0.18 | .43 |
| Doctorate degree | -0.01 | .95 |
| *(Age 🡪 Opinions on vaccination in general)* |  |  |
| 50-75+ | 1 |  |
| 18-49 | -0.37 | **<.001** |
| *(Financial deprivation 🡪 Opinions on vaccination in general)* |  |  |
| No | 1 |  |
| Yes | -0.05 | .62 |
| *(One or more chronic disease 🡪 Opinion on vaccination in general)* |  |  |
| No | 1 |  |
| Yes | 0.07 | .44 |
| *(Education level 🡪 Opinion on vaccination in general)* |  |  |
| No diploma | 1 |  |
| Lower secondary school certificate | 0.13 | .48 |
| Upper secondary school certificate | 0.21 | .28 |
| Bachelor’s degree | 0.17 | .40 |
| Master’s degree | 0.23 | .29 |
| Doctorate degree | 0.59 | **.004** |
| *(Age 🡪 Trust in the healthcare system)* |  |  |
| 50-75+ | 1 |  |
| 18-49 | -0.13 | **.006** |
| *(Financial deprivation 🡪 Trust in the healthcare system)* |  |  |
| No | 1 |  |
| Yes | -0.16 | **.008** |
| *(One or more chronic disease 🡪 Trust in the healthcare system)* |  |  |
| No | 1 |  |
| Yes | 0.06 | .28 |
| *(Education level 🡪 Trust in the healthcare system)* |  |  |
| No diploma | 1 |  |
| Lower secondary school certificate | -0.01 | .96 |
| Upper secondary school certificate | 0.04 | .74 |
| Bachelor’s degree | 0.09 | .46 |
| Master’s degree | 0.21 | .11 |
| Doctorate degree | 0.21 | .06 |

- **age<50**

| **Model pathways** | **Estimated** | **p-value** |
| --- | --- | --- |
| *(Gender 🡪 Opinions on hepatitis B vaccination)* |  |  |
| Men | 1 |  |
| Women | -0.13 | .06 |
| *(Financial deprivation 🡪 Opinions on hepatitis B vaccination)* |  |  |
| No | 1 |  |
| Yes | -0.07 | .32 |
| *(One or more chronic disease 🡪 Opinions on hepatitis B vaccination)* |  |  |
| No | 1 |  |
| Yes | 0.10 | .16 |
| *(Education level 🡪 Opinions on hepatitis B vaccination)* |  |  |
| No diploma | 1 |  |
| Lower secondary school certificate | 0.13 | .34 |
| Upper secondary school certificate | -0.09 | .50 |
| Bachelor’s degree | -0.08 | .56 |
| Master’s degree | 0.07 | .64 |
| Doctorate degree | -0.06 | .71 |
| *(Gender 🡪 Opinions on vaccination in general)* |  |  |
| Men | 1 |  |
| Women | -0.11 | .14 |
| *(Financial deprivation 🡪 Opinions on vaccination in general)* |  |  |
| No | 1 |  |
| Yes | -0.15 | .06 |
| *(One or more chronic disease 🡪 Opinion on vaccination in general)* |  |  |
| No | 1 |  |
| Yes | 0.06 | .48 |
| *(Education level 🡪 Opinion on vaccination in general)* |  |  |
| No diploma | 1 |  |
| Lower secondary school certificate | 0.10 | .58 |
| Upper secondary school certificate | 0.13 | .47 |
| Bachelor’s degree | 0.07 | .71 |
| Master’s degree | 0.28 | .16 |
| Doctorate degree | 0.41 | **.03** |
| *(Gender 🡪 Trust in the healthcare system)* |  |  |
| Men | 1 |  |
| Women | -0.11 | **.**10 |
| *(Financial deprivation 🡪 Trust in the healthcare system)* |  |  |
| No | 1 |  |
| Yes | -0.12 | .09 |
| *(One or more chronic disease 🡪 Trust in the healthcare system)* |  |  |
| No | 1 |  |
| Yes | 0.03 | .64 |
| *(Education level 🡪 Trust in the healthcare system)* |  |  |
| No diploma | 1 |  |
| Lower secondary school certificate | -0.22 | .12 |
| Upper secondary school certificate | -0.07 | .62 |
| Bachelor’s degree | -0.08 | .59 |
| Master’s degree | 0.11 | .51 |
| Doctorate degree | 0.07 | .65 |

- **age≥50**

| **Model pathways** | **Estimated** | **p-value** |
| --- | --- | --- |
| *(Gender 🡪 Opinions on hepatitis B vaccination)* |  |  |
| Men | 1 |  |
| Women | -0.24 | **<.001** |
| *(Financial deprivation 🡪 Opinions on hepatitis B vaccination)* |  |  |
| No | 1 |  |
| Yes | 0.22 | **.004** |
| *(One or more chronic disease 🡪 Opinions on hepatitis B vaccination)* |  |  |
| No | 1 |  |
| Yes | 0.06 | .40 |
| *(Education level 🡪 Opinions on hepatitis B vaccination)* |  |  |
| No diploma | 1 |  |
| Lower secondary school certificate | -0.14 | .40 |
| Upper secondary school certificate | -0.22 | .21 |
| Bachelor’s degree | -0.06 | .72 |
| Master’s degree | -0.01 | .98 |
| Doctorate degree | -0.01 | .94 |
| *(Gender 🡪 Opinions on vaccination in general)* |  |  |
| Men | 1 |  |
| Women | -0.15 | .06 |
| *(Financial deprivation 🡪 Opinions on vaccination in general)* |  |  |
| No | 1 |  |
| Yes | -0.15 | .09 |
| *(One or more chronic disease 🡪 Opinion on vaccination in general)* |  |  |
| No | 1 |  |
| Yes | 0.09 | .28 |
| *(Education level 🡪 Opinion on vaccination in general)* |  |  |
| No diploma | 1 |  |
| Lower secondary school certificate | 0.20 | .23 |
| Upper secondary school certificate | 0.33 | .07 |
| Bachelor’s degree | 0.18 | .30 |
| Master’s degree | 0.20 | .35 |
| Doctorate degree | 0.58 | **.004** |
| *(Gender 🡪 Trust in the healthcare system)* |  |  |
| Men | 1 |  |
| Women | -0.16 | **.005** |
| *(Financial deprivation 🡪 Trust in the healthcare system)* |  |  |
| No | 1 |  |
| Yes | -0.19 | **.002** |
| *(One or more chronic disease 🡪 Trust in the healthcare system)* |  |  |
| No | 1 |  |
| Yes | 0.18 | **.002** |
| *(Education level 🡪 Trust in the healthcare system)* |  |  |
| No diploma | 1 |  |
| Lower secondary school certificate | -0.10 | .31 |
| Upper secondary school certificate | -0.14 | .19 |
| Bachelor’s degree | 0.06 | .61 |
| Master’s degree | 0.04 | .75 |
| Doctorate degree | 0.07 | .61 |

- **No financial difficulties**

| **Model pathways** | **Estimated** | **p-value** |
| --- | --- | --- |
| *(Gender 🡪 Opinions on hepatitis B vaccination)* |  |  |
| Men | 1 |  |
| Women | -0.14 | .06 |
| *(Age 🡪 Opinions on hepatitis B vaccination)* |  |  |
| 50-75+ | 1 |  |
| 18-49 | 0.36 | **<.001** |
| *(One or more chronic disease 🡪 Opinions on hepatitis B vaccination)* |  |  |
| No | 1 |  |
| Yes | 0.11 | .15 |
| *(Education level 🡪 Opinions on hepatitis B vaccination)* |  |  |
| No diploma | 1 |  |
| Lower secondary school certificate | -0.06 | .73 |
| Upper secondary school certificate | -0.22 | .21 |
| Bachelor’s degree | -0.09 | .63 |
| Master’s degree | -0.05 | .81 |
| Doctorate degree | -0.03 | .89 |
| *(Gender 🡪 Opinions on vaccination in general)* |  |  |
| Men | 1 |  |
| Women | -0.08 | .27 |
| *(Age 🡪 Opinions on vaccination in general)* |  |  |
| 50-75+ | 1 |  |
| 18-49 | -0.36 | **<.001** |
| *(One or more chronic disease 🡪 Opinion on vaccination in general)* |  |  |
| No | 1 |  |
| Yes | 0.07 | .37 |
| *(Education level 🡪 Opinion on vaccination in general)* |  |  |
| No diploma | 1 |  |
| Lower secondary school certificate | 0.15 | .34 |
| Upper secondary school certificate | 0.17 | .31 |
| Bachelor’s degree | 0.13 | .42 |
| Master’s degree | 0.17 | .33 |
| Doctorate degree | 0.46 | **.008** |
| *(Gender 🡪 Trust in the healthcare system)* |  |  |
| Men | 1 |  |
| Women | -0.17 | **<.001** |
| *(Age 🡪 Trust in the healthcare system)* |  |  |
| 50-75+ | 1 |  |
| 18-49 | -0.13 | **.008** |
| *(One or more chronic disease 🡪 Trust in the healthcare system)* |  |  |
| No | 1 |  |
| Yes | 0.14 | **.006** |
| *(Education level 🡪 Trust in the healthcare system)* |  |  |
| No diploma | 1 |  |
| Lower secondary school certificate | -0.02 | .83 |
| Upper secondary school certificate | 0.06 | .54 |
| Bachelor’s degree | 0.14 | .18 |
| Master’s degree | 0.26 | **.02** |
| Doctorate degree | 0.26 | **.01** |

- **Financial difficulties**

| **Model pathways** | **Estimated** | **p-value** |
| --- | --- | --- |
| *(Gender 🡪 Opinions on hepatitis B vaccination)* |  |  |
| Men | 1 |  |
| Women | -0.26 | **.001** |
| *(Age 🡪 Opinions on hepatitis B vaccination)* |  |  |
| 50-75+ | 1 |  |
| 18-49 | 0.14 | **.10** |
| *(One or more chronic disease 🡪 Opinions on hepatitis B vaccination)* |  |  |
| No | 1 |  |
| Yes | 0.06 | .42 |
| *(Education level 🡪 Opinions on hepatitis B vaccination)* |  |  |
| No diploma | 1 |  |
| Lower secondary school certificate | -0.00 | .99 |
| Upper secondary school certificate | -0.10 | .53 |
| Bachelor’s degree | -0.08 | .66 |
| Master’s degree | 0.16 | .48 |
| Doctorate degree | -0.20 | .35 |
| *(Gender 🡪 Opinions on vaccination in general)* |  |  |
| Men | 1 |  |
| Women | -0.21 | **.02** |
| *(Age 🡪 Opinions on vaccination in general)* |  |  |
| 50-75+ | 1 |  |
| 18-49 | -0.30 | **.002** |
| *(One or more chronic disease 🡪 Opinion on vaccination in general)* |  |  |
| No | 1 |  |
| Yes | 0.11 | .22 |
| *(Education level 🡪 Opinion on vaccination in general)* |  |  |
| No diploma | 1 |  |
| Lower secondary school certificate | 0.16 | .38 |
| Upper secondary school certificate | 0.29 | .15 |
| Bachelor’s degree | 0.13 | .50 |
| Master’s degree | 0.52 | .048 |
| Doctorate degree | 0.60 | **.01** |
| *(Gender 🡪 Trust in the healthcare system)* |  |  |
| Men | 1 |  |
| Women | -0.09 | .12 |
| *(Age 🡪 Trust in the healthcare system)* |  |  |
| 50-75+ | 1 |  |
| 18-49 | -0.10 | .11 |
| *(One or more chronic disease 🡪 Trust in the healthcare system)* |  |  |
| No | 1 |  |
| Yes | 0.07 | .28 |
| *(Education level 🡪 Trust in the healthcare system)* |  |  |
| No diploma | 1 |  |
| Lower secondary school certificate | -0.27 | **.01** |
| Upper secondary school certificate | -0.26 | **.03** |
| Bachelor’s degree | -0.18 | .13 |
| Master’s degree | -0.12 | .45 |
| Doctorate degree | -0.22 | .13 |
